# Comparative Molecular Biology Approaches for the Production of Poliovirus Virus-Like Particles Using *Pichia pastoris*

**DOI:** 10.1128/mSphere.00838-19

**Published:** 2020-03-11

**Authors:** Lee Sherry, Keith Grehan, Joseph S. Snowden, Michael L. Knight, Oluwapelumi O. Adeyemi, David J. Rowlands, Nicola J. Stonehouse

**Affiliations:** a School of Molecular and Cellular Biology, Faculty of Biological Sciences, University of Leeds, Leeds, United Kingdom; b Sir William Dunn School of Pathology, University of Oxford, Oxford, United Kingdom; c Department of Medical Microbiology and Parasitology, Faculty of Basic Medical Sciences, College of Health Sciences, University of Ilorin, Ilorin, Nigeria; University of Zurich

**Keywords:** enterovirus, poliovirus, virus-like particle, *Pichia pastoris*

## Abstract

For enteroviruses such as poliovirus (PV), empty capsids, which are antigenically indistinguishable from mature virions, are produced naturally during viral infection. The production of such capsids recombinantly, in heterologous systems such as yeast, have great potential as virus-like particle (VLP) vaccine candidates. Here, using PV as an exemplar, we show the production of VLPs in Pichia pastoris by coexpression of the structural precursor protein P1 and the viral protease 3CD. The level of expression of the potentially cytotoxic protease relative to that of the P1 precursor was modulated by three different approaches: expression of the P1 precursor and protease from different transcription units, separation of the P1 and protease proteins using the *Thosea asigna* virus (TaV) 2A translation interruption sequence, or separation of the P1 and protease-coding sequences by an internal ribosome entry site sequence from *Rhopalosiphum padi* virus (RhPV). We also investigate the antigenicity of VLPs containing previously characterized mutations when produced in *Pichia*. Finally, using transmission electron microscopy and two-dimensional classification, we show that *Pichia*-derived VLPs exhibited the classical icosahedral capsid structure displayed by enteroviruses.

**IMPORTANCE** Although the current poliovirus immunization program has been extremely successful in reducing the number of cases of paralytic polio worldwide, now more cases are caused by vaccine-derived polioviruses than by wild poliovirus. Switching to inactivated poliovirus vaccines will reduce this over time; however, their production requires the growth of large amounts of virus. This biosafety concern can be addressed by producing just the virus capsid. The capsid serves to protect the genetic material, which causes disease when introduced into a cell. Therefore, empty capsids (virus-like particles [VLPs]), which lack the viral RNA genome, are safe both to make and to use. We exploit yeast as a versatile model expression system to produce VLPs, and here we specifically highlight the potential of this system to supply next-generation poliovirus vaccines to secure a polio-free world for the future.

## Abstract (precis)

Although the current poliovirus immunization program has been extremely successful in reducing the number of cases of paralytic polio worldwide, now more cases are caused by vaccine-derived polioviruses than by wild poliovirus. Switching to inactivated poliovirus vaccines will reduce this over time; however, their production requires the growth of large amounts of virus. This biosafety concern can be addressed by producing just the virus capsid. The capsid serves to protect the genetic material, which causes disease when introduced into a cell. Therefore, empty capsids (virus-like particles [VLPs]), which lack the viral RNA genome, are safe both to make and to use. We exploit yeast as a versatile model expression system to produce VLPs, and here we specifically highlight the potential of this system to supply next-generation poliovirus vaccines to secure a polio-free world for the future.

## INTRODUCTION

Enteroviruses are responsible for a number of human and animal diseases, with poliovirus (PV) being the best-characterized member. PV is the causative agent of poliomyelitis, a devastating paralytic and sometimes fatal infection that was responsible for global epidemics in the last century. However, since the introduction of the Global Polio Eradication Initiative in 1988, there has been a >99% reduction in the number of cases worldwide, with wild-type (wt) poliovirus now only endemic to Afghanistan, Pakistan, and Nigeria (http://polioeradication.org/polio-today/polio-now/). This reduction is primarily attributable to the use of two highly effective vaccines, the live-attenuated oral PV vaccine (OPV) and the inactivated PV vaccine (IPV), both of which target all three PV serotypes (PV-1, PV-2, and PV-3). wt PV-2 has been declared globally eradicated, as none has been isolated since 1999 (1). However, despite the success of these vaccines, there are significant safety concerns for their continued use in a “polio-free” world to guard against the potential catastrophe that would result from the reintroduction of the virus.

Due to the genetic instability of OPV, the attenuated virus can quickly revert to virulence, leading to vaccine-associated paralytic poliomyelitis (VAPP), especially in areas of low vaccine coverage, leading to circulating vaccine-derived PV (cVDPV) (2). Indeed, cVDPV cases now outnumber wt PV-derived cases worldwide (http://polioeradication.org/polio-today/polio-now/). Additionally, individuals with undiagnosed compromised immune systems may become chronic carriers of cVDPV postvaccination, acting as reservoirs capable of reintroducing PV to the environment (2, 3). Although IPV offers effective protection against polio disease, it does not prevent the replication of the virus in infected individuals and, therefore, cannot halt further transmission of the virus within a population (4). Furthermore, the production of IPV requires the growth of large amounts of highly concentrated infectious virus, which could be catastrophic in the event of accidental release (https://www.ecdc.europa.eu/en/threats-and-outbreaks).

**FIG 1:**
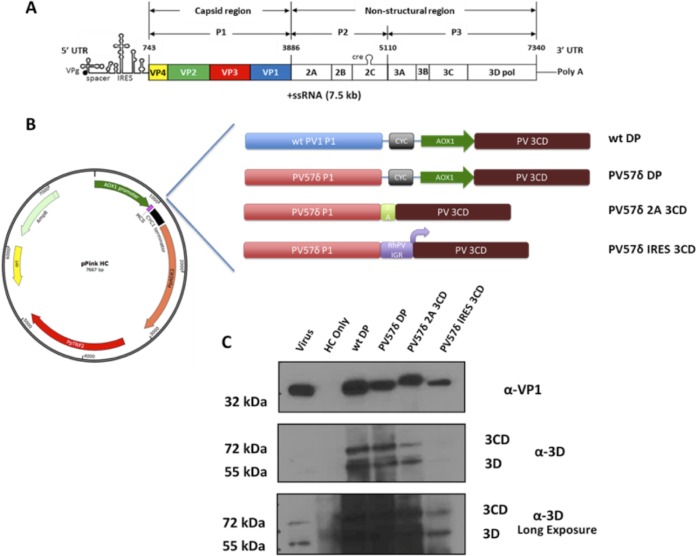
Schematic of the poliovirus genome, *Pichia* expression systems, and correct processing of PV P1. (A) Schematic of the poliovirus genome highlighting the P1 structural region and the nonstructural proteins, including the viral protease 3CD. UTR, untranslated region; ssRNA, single-stranded RNA. (B) Schematic of the pPink HC plasmid and the position in which the different expression constructs were cloned. (C) Immunoblot for PV VP1 and 3D. The virus control sample was prepared from HeLa cell lysates 8 h postinfection. Yeast samples were collected 48 h postinduction and lysed using 0.1 M NaOH. All samples were boiled in 2× Laemmli buffer and separated by SDS-PAGE prior to analysis by immunoblotting using mouse monoclonal α-VP1 and rabbit monoclonal α-3D antibody. Two exposures of the same α-3D immunoblot are shown to aid interpretation.

PV is a positive-sense RNA virus, belonging to the species *Enterovirus C*, with a 7.5-kb genome that contains 2 overlapping reading frames. The recently discovered small open reading frame (ORF) of unknown function has been shown to be important for replication in *ex vivo* human enteroids (5). The major ORF (Fig. 1A) is translated as a single large polyprotein that is proteolytically cleaved into the mature viral proteins (5, 6). The viral protease 3C and its precursor, 3CD, play key roles in viral replication. 3C is responsible for the processing of the P2 and P3 regions of the viral polyprotein, whereas 3CD has been shown *in vitro* to be responsible primarily for cleavage of the structural precursor protein P1 (7, 8). Additionally, 3CD also has been shown to have an integral role in priming the viral RNA for replication by the viral polymerase 3D (9, 10). P1 is processed to produce the capsid proteins VP0, VP3, and VP1, with the subsequent cleavage of VP0 into VP2 and VP4 thought to be driven by the encapsidation of the viral genome (11, 12). Mature PV virions contain 60 copies of each of VP1 to VP4, forming an ∼30-nm icosahedral capsid (13). In addition, PV infection also leads to the production of empty capsids (ECs), which lack the viral genome but are otherwise identical to the mature virions, except that they contain uncleaved VP0 (14). Recombinantly expressed ECs could be developed as a virus-like particle (VLP) vaccine to replace the current vaccines against PV. However, wt ECs are inherently unstable, resulting in a conformational expansion of the particles and conversion from native antigenicity (termed D antigen) to the nonnative form (termed C antigen) (14, 15). This conformational switch is of particular importance as the nonnative antigen is a poor inducer of neutralizing antibodies and, therefore, is incapable of providing a long-lasting, protective immune response against PV (15–17). Therefore, any attempts to produce PV VLPs must also attempt to ensure the particles maintain the D antigenic form.

PV VLPs have been produced in a number of different expression systems, including mammalian, plant, and insect cells (18–22). Although the secretion of VLPs from insect cells is an attractive option, each of these systems can incur high production costs, making them less accessible for lower- to middle-income countries (LMICs). Yeast is a heterologous expression system with potential for technology transfer to LMICs, as it is inexpensive in terms of medium costs and infrastructure requirements. Additionally, yeast expression is currently licensed for the production of human vaccines against two viral pathogens, hepatitis B virus and human papillomavirus (23–25).

Using P1 sequences from the wt and a previously characterized mutant, we show that PV VLPs can be produced in the yeast Pichia pastoris via three different expression constructs. The expression of the potentially cytotoxic 3CD is modulated using different molecular biology approaches. The VLPs produced from each construct show physical properties similar to those of the ECs produced naturally during infection. Additionally, through two-dimensional (2D) class averaging of electron microscopy data, we show that PV VLPs produced from each expression cassette maintain the classic icosahedral morphology associated with picornavirus capsids, highlighting the potential of Pichia pastoris as a model system for the production of enterovirus VLPs.

## RESULTS

### Correct processing of PV P1 in Pichia pastoris.

To determine the feasibility of producing enterovirus VLPs in *Pichia*, using PV as an exemplar, we employed three different expression cassettes, as described for Fig. 1B, each containing the P1 region together with the viral protease. Expression should result in the production of VP0, VP1, and VP3 and assembly into VLPs. A dual promoter expression system previously was used to produce VLPs for other enteroviruses, such as coxsackie A6, coxsackie A16, and enterovirus A71 (26–28). We employed this approach here for the wt and for a previously described capsid mutant of PV-1 Mahoney (PV57δ), which we have shown to increase D antigen stability in mammalian cell culture (29). Due to concerns over the potential toxicity of the viral protease 3CD, we modulated the level of 3CD expression to investigate its impact on VLP production (30). To this end, we designed two other constructs for comparative analysis alongside the dual promoter, using PV57δ (Fig. 1B).

To modulate the expression of 3CD, we introduced either the *Thosea asigna* virus (TaV) 2A peptide sequence between the end of P1 and the start of 3CD or the intergenic region internal ribosome entry site (IGR IRES; referred to as RhPV IRES) from *Rhopalosiphum padi* virus (RhPV) between P1 and 3CD. TaV 2A peptide has one of the most efficient cleavage sequences, which should lead to a ratio of P1 to 3CD between 1:1 and 2:1, depending on the efficiency of translation reinitiation (31). The introduction of the RhPV IRES should have a more pronounced impact on the levels of 3CD, as IRES-mediated translation has been shown to be 5- to 20-fold lower than cap-driven translation (32).

Following transformation, *Pichia* colonies were tested for expression levels. Following 48 h of induction, samples from small-scale test expression were indirectly analyzed for the correct processing of P1 polyprotein using an anti-VP1 antibody (Fig. 1C). The anti-VP1 immunoblot showed that material derived from all three expression cassettes correctly processed P1, resulting in the appearance of an immunoreactive protein similar in size to the VP1 produced in PV-1 Mahoney-infected HeLa cell lysate, while the vector-only *Pichia* control (HC only) showed no reactivity. The VP1 band for PV57δ-2A-3CD showed a small mobility shift, reflecting the 18-amino-acid extension to the C terminus of VP1. These lysates were also probed with anti-3D, which showed two major bands, at approximately 72 and 55 kDa, corresponding to the expected sizes of 3CD and 3D (Fig. 1C). The anti-3D immunoblot showed similar levels of 3CD expression between the dual promoter and 2A expression cassettes; however, as expected, the IRES-mediated 3CD expression was much lower.

### Material produced in Pichia pastoris sedimented similarly to PV-1 empty capsids.

**FIG 2:**
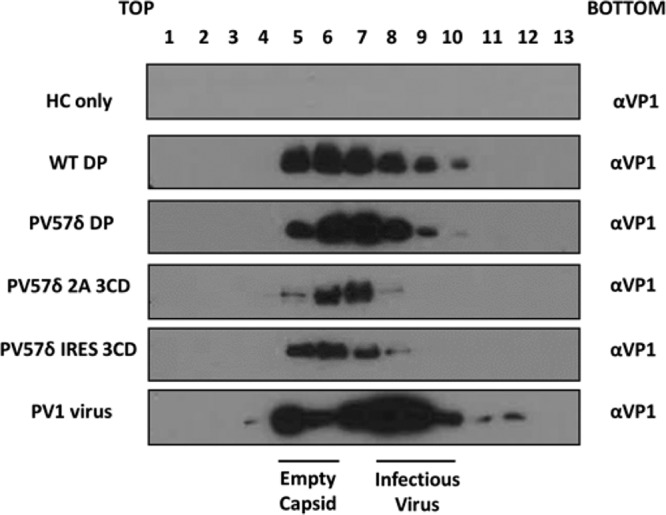
Purification of virions and VLPs. All samples were purified by ultracentrifugation and fractioned from top to bottom in 1-ml aliquots. A 12-μl sample from each fraction then was taken and boiled in 5× Laemmli buffer and separated by SDS-PAGE prior to analysis by immunoblotting using mouse monoclonal α-VP1. Shown is a representative example of three separate experiments for each construct.

The best-expressing *Pichia* clones for each expression system then were induced using 0.5% methanol in a 200-ml culture volume, and cell pellets collected 48 h postinduction for purification. To determine whether each of the different expression systems produced higher-order structures, the cell pellets were homogenized at ∼275 MPa and subjected to multistep centrifugal purification, culminating in separation through 15 to 45% sucrose gradients. Gradient fractions then were analyzed for the presence of VP1 by immunoblot assay (Fig. 2). As expected, the HC vector-only sample was not positive for VP1, whereas the expression cassettes for wt DP, PV57δ DP, and PV57δ-IRES-3CD produced material that peaked in fraction 6. For PV57δ-2A-3CD, the peak was slightly further down the gradient (in fraction 7). As a control, PV-1 Mahoney virus also was purified through a 15 to 45% sucrose gradient and probed for VP-1 by immunoblotting, and it showed two distinct populations, as expected (29), corresponding to peaks for ECs in fractions 4 and 5 and infectious virions in fractions 8 and 9.

### *Pichia*-derived VLPs are structurally similar to infectious PV.

**FIG 3:**
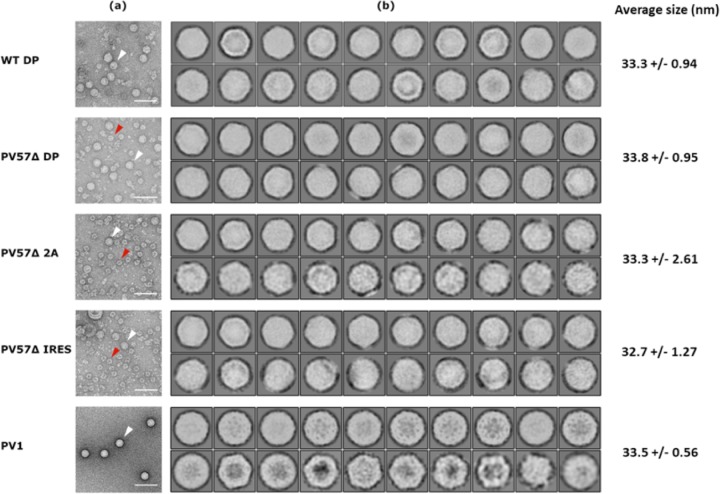
Electron microscopy and 2D class averages of PV VLPs. (a) Representative micrographs from different preparations of PV VLPs and infectious PV (PV-1). White arrowheads indicate ∼33-nm-diameter particles. Red arrowheads indicate smaller particles (∼22-nm diameter). Scale bar shows 100 nm. (b) 2D class averages from the twenty most populated classes of ∼33-nm-diameter particles in each data set, following removal of the smaller particles. Class averages are shown with a sigma contrast of 3. Average particle size, *n* = 20 for each preparation.

Although the density gradient purification suggested that the viral protein produced assembled as VLPs, we sought to confirm this by electron microscopy (EM) and 2D class averaging. Figure 3a shows a representative micrograph of *Pichia*-derived VLPs from each expression cassette compared with gradient-purified infectious PV. The negative-stain EM images show *Pichia*-derived VLPs to be of morphology similar to that of PV particles produced by mammalian cells. The VLPs were similar in diameter across both wt and PV57δ VLPs as well as between the different expression cassettes (33.4 ± 1.40 nm [*n *= 20]). The diameters of the *Pichia*-derived VLPs were very similar to the diameter of infectious PV virions (33.5 ± 0.56 nm [*n *= 20]). In the micrographs of PV57δ samples, small particles, depicted by the red arrows, far outnumbered the larger ∼33-nm particles, suggesting an assembly defect for this mutant.

The larger particles (white arrows) then were analyzed by 2D classification (Fig. 3b). Class averages were highly indicative of icosahedral structure for both the *Pichia*-derived VLPs and PV virions. This analysis also showed the VLPs tended to be more angular in shape than the PV virions, indicating that they are in the nonnative conformation. Native (D antigenic) particles are known to be smoother in appearance, as seen in the class averages for PV virions (33, 34). However, the classes show variation in both the size and shape for all expression cassettes, suggesting a level of heterogeneity in the *Pichia*-derived VLPs. Interestingly, the addition of 2A to the C terminus of VP1 did not lead to overall changes in particle morphology that could be determined by negative-stain EM.

### *Pichia*-derived VLPs did not package substantial levels of nucleic acid.

To further determine that the VLPs produced in *Pichia* were empty and had not packaged cognate mRNA or any nonspecific yeast RNA, the peak fraction for each expression cassette was pelleted at 151,000 × *g* and resuspended in phosphate-buffered saline (PBS) and 20 mM EDTA prior to analysis at absorbance wavelengths of 260 nm and 280 nm. The 260/280 nm ratios shown in Table 1 indicate that the level of nucleic acid present in the VLP samples was very low. This suggests that the VLPs produced in *Pichia* are reminiscent of ECs produced during mammalian cell infection, which contain no nucleic acid (35).

**TABLE 1:** Absorbance at 260/280 nm^*a*^

Sample	Absorbance at 260/280 nm
PV-1 infectious virus	1.54
HC only	0.80
WT DP	1.04
PV57δ DP	0.75
PV57δ 2A 3CD	0.81
PV57δ IRES 3CD	0.79

a Peak gradient fractions were pelleted and resuspended in PBS plus 20mM EDTA. Samples were analyzed for absorbance at wavelengths of both 260 and 280 nm using NanoDrop One (Thermo Scientific).

### PV57δ shows no improvement in D antigen content.

Following the observation that *Pichia*-derived VLPs showed characteristics similar to those of ECs, we next determined their antigenic character by enzyme-linked immunosorbent assay (ELISA). We analyzed gradient fractions using a standard protocol, as established by the National Institute for Biological Standards and Control (NIBSC), using the current inactivated vaccine (BRP) as a positive control (36).

**FIG 4:**
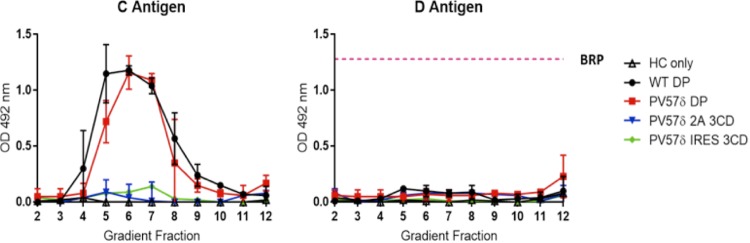
Antigenicity of *Pichia*-derived VLPs. Shown are the antigenicity of *Pichia*-derived PV-1 wt and PV57δ VLPs and the reactivity of gradient fractions with MAb 1588 (C antigen) and MAb 234 (D antigen) in ELISA. The pink dashed line represents the positive control, BRP, for the D antigen ELISA. The optical density at 492 nm (OD 492 nm) is represented in arbitrary units (*n* = 2).

Reagents specific to C antigen revealed that both wt DP and PV57δ DP VLPs produced large amounts of C antigen, whereas the PV57δ-2A-3CD and PV57δ-IRES-3CD VLPs were below the threshold of detection (Fig. 4). This suggests that despite VLPs being processed correctly and detectable by immunoblotting, these two expression systems are less efficient for VLP production than the dual promoter system. However, neither the wt nor any of the PV57δ VLPs contained detectable D antigen in this assay. As expected, the HC-only control gradient fractions were negative for both D and C antigenic forms.

## DISCUSSION

As we move toward the eradication of poliovirus, the number of cVDPV cases is now outnumbering the number of paralytic polio cases caused by wt PV (http://polioeradication.org/polio-today/polio-now/). However, both OPV and IPV require the production of large amounts of infectious virus, which raises biosafety concerns around the potential for the reintroduction of PV to the environment (1, 4). Therefore, as we edge closer to a polio-free world, we need to consider alternative vaccine production strategies to ensure control of PV is maintained. One solution is to produce a virus-free VLP vaccine using a heterologous expression system. This approach has been successful for the production of PV VLPs in both plant and insect cell systems, but both of these require specialist equipment, which increases the cost of production, making it difficult to translate these production methods to LMICs (18–20). Yeast offers an alternative heterologous expression system that can produce levels of protein production similar to those of mammalian cells (e.g., 400 mg/liter compared to 500 mg/liter from mammalian cells [37]) but at a fraction of the cost and with the added advantages of greater speed and ease of scalability (37–39). Therefore, we looked to build on the previous work of Rombaut and Jore by producing PV VLPs in yeast to determine if this is a viable expression system for future enterovirus vaccine production, especially in light of the recent emergence of the neurovirulent enterovirus D68 as well as the increasing cases of enterovirus A71 and coxsackievirus A16, the etiological agents of hand, foot, and mouth disease (40–42).

First, we investigated the production of PV VLPs using a range of expression cassettes. The dual promoter expression cassette was used previously for the production of other picornavirus VLPs, while the introduction of TaV 2A and RhPV IRES was used to modulate the expression of 3CD (26–28). 3C protease previously was shown to have a toxic effect on cell viability, whereas 3CD was shown to be the active protease in P1 processing and should be less toxic than 3C (8, 30). Therefore, in an attempt to reduce protease toxicity, we introduced a previously characterized noncleavable 3CD into our expression constructs (43). However, as shown in Fig. 1C, there is clear cleavage of 3CD, suggesting that the noncleavable phenotype is expression system dependent. Presumably, a host protease is responsible for the 3CD cleavage seen, which occurs at a sequence close to the natural cleavage site. It would be interesting to fully ablate this cleavage event in *Pichia* or introduce mutations into 3C^pro^ to reduce toxicity, as shown previously for foot-and-mouth disease virus, and determine the impact of these modifications on VLP production (44). We demonstrated that all three expression cassettes led to the correct processing of P1 through the detection of VP-1 using monoclonal antibody (MAb) 8650. This antibody also can detect full-length P1 and other processing intermediates; however, we detected no structural precursors by immunoblotting, suggesting good P1 processing efficiency in each expression cassette. Additionally, we also showed that both the TaV 2A and RhPV IRES were able to control 3CD translation in *Pichia*. These could be important tools for the production of VLPs from other virus families, such as flaviviruses or orthomyxoviruses, which may require differential expression of constructs required for protein maturation (45, 46).

The production of VLPs from each expression cassette was determined by density gradient centrifugation and further confirmed by negative-stain EM. The VLPs all showed rates of sedimentation similar to that of the ECs produced from virus infection of mammalian cells. Interestingly, the peak produced from the PV57δ-2A-3CD VLPs was detected one fraction further down the gradient than the other VLPs. These VLPs were assembled with an additional 20-amino-acid extension present on the C terminus of VP1, suggesting the 2A peptide has an impact on the sedimentation rate of VLPs.

To ensure that no host RNA or cognate mRNA was being nonselectively packaged into the VLPs, we used the 260/280-nm absorbance ratio to estimate the level of nucleic acid present in the purified VLPs. The ratio of nucleic acid to protein was far lower than that in the purified infectious virus and was similar to the levels of nucleic acid found in equivalent fractions in the vector-only purified yeast lysate, suggesting that the VLPs did not encapsidate substantial quantities of nonspecific nucleic acid.

2D particle analysis showed that all the expression cassettes produced icosahedral particles of ∼33 nm in diameter. Given that negative staining leads to an overestimation of particle size, these are very similar to the sizes of virions produced from natural infection (47). The micrographs also highlighted a number of smaller particles purified along with PV57δ VLPs, whereas these contaminating particles were rarely seen in the wt VLP purifications. These small particles may represent assembly defects for the PV57δ mutant, leading to partially assembled particles. If so, these defects could be alleviated by the coexpression of HSP-90, which previously was shown to be beneficial for PV capsid assembly (48). Alternatively, these particles could be contaminants, which, for example, could be derived from yeast chaperones or enzyme complexes that copurify with PV57δ for unknown reasons.

Unfortunately, the VLPs produced in this study were in the nonnative conformation, as highlighted by ELISA (Fig. 4), and the clear conformational difference between the *Pichia*-derived VLPs and the PV virions was apparent by 2D classification (Fig. 3b). Although this was expected for the wt sequence, it was surprising for PV57δ. The PV57δ mutant previously was shown to produce ECs in both C and D antigenic conformations in mammalian cells by immunoprecipitation (29). It is clear that the ELISA may not detect lower levels of antigen, as illustrated by no detection of either PV57δ-2A-3CD or PV57δ-IRES-3CD here. These data also suggest that the alternative, dual promoter expression cassette should be the primary choice for the production of other enterovirus VLPs. Although we cannot be certain that the *Pichia*-derived VLPs produced here would not lead to a protective immune response, the observation that these are positive for C antigen suggests that they are poor inducers of neutralizing antibodies and, therefore, are unlikely to induce protective immune responses (15–17). The lack of pocket factor or the incorporation of a suboptimal molecule also could be responsible for the dominance of the C conformation. Pocket factor has been identified as a small lipid moiety that binds in a hydrophobic cleft in VP1 (47). This molecule contributes to maintaining capsid stability and is displaced during receptor binding and consequent conformational changes associated with the cell entry process. Capsids produced in mammalian cells have a natural pocket factor; however, it is unclear whether VLPs produced in heterologous expression systems bind a cofactor that occupies the pocket. Structural analysis by cryo-EM of plant-derived PV VLPs failed to demonstrate the presence of pocket factor, suggesting that pocket-binding lipids can be expression system dependent (18). Additionally, Rombaut and Jore (40) found that wt PV VLPs produced in Saccharomyces cerevisiae required the addition of pocket binding compounds to maintain the native conformation, suggesting either the absence of pocket factor or the incorporation of a pocket factor with low binding affinity, which was easily displaced through the addition of pocket binding compounds.

In conclusion, we believe we have shown that Pichia pastoris is a viable expression system for large-scale production of PV VLPs, which share characteristics with ECs produced from mammalian cells. In accordance with other studies, we have shown that a dual promoter expression system is the most efficient way of producing picornavirus VLPs. Additionally, we also demonstrated translational control over protein expression levels through the use of molecular biology approaches, utilizing the TaV 2A peptide and the RhPV IRES. Although the PV57δ VLPs used here were shown to have stability superior to that of the wt when produced in a mammalian system, the stabilization did not extend to particles expressed in yeast, possibly for the reasons outlined above. However, in preliminary experiments using the thermostable mutants previously characterized by Fox et al. (18, 20, 49), we have shown *Pichia* to be capable of efficiently producing D antigen. Therefore, we believe that this system has the potential to produce a VLP vaccine not only for a polio-free world but also as a model system for enterovirus VLP vaccine production.

## MATERIALS AND METHODS

### Cells and viruses.

HeLa cells were obtained from the National Institute of Biological Standards and Controls (NIBSC), UK. PichiaPink yeast strain one (Invitrogen, USA) was grown according to the instructions of the manufacturer. The cDNA of wt PV-1 (Mahoney strain) used in this study was sourced from Bert Semler, University of California, and was cloned downstream of a T7 RNA promoter to allow *in vitro* RNA synthesis. A hammerhead ribozyme was included at the 5′ end, resulting in the production of authentic infectious PV-1 RNA (10). To initiate infection, L cells (which lack the PV receptor CD155) were transfected with PV-1 RNA, and the resulting viruses were propagated in HeLa cells.

### Vector construction.

The P1 genes of PV-1 Mahoney and PV57δ were optimized according to P. pastoris preferred codon usage, synthesized (GeneART), and inserted into the pPink-HC expression vector multiple cloning site (MCS) using EcoRI and FseI (New England Biolabs [NEB]). The 3CD gene was also codon optimized for P. pastoris and designed to include a noncleavable sequence to reduce the potential toxic effects of 3C in a heterologous expression system (43). The 3CD gene then was cloned into the pPink-HC expression vector MCS using EcoRI and FseI (NEB). For the coexpression of P1 and 3CD, a number of approaches were taken. For the dual promoter constructs, the region from position 1 of the 3CD pPink-HC to position 1285 was amplified by PCR, with the primers inserting a SacII restriction site at both the 5′ and 3′ ends of the product. The P1 expression plasmid was linearized by SacII (NEB), and then the 3CD PCR product was inserted into SacII-linearized P1 plasmid. Clones then were screened for directionality, ensuring the promoters for each protein were in the correct orientation. The PV57δ-2A-3CD plasmid was constructed through a series of overlapping PCRs. Briefly, the TaV 2A peptide sequence was inserted upstream of 3CD through overlapping PCR. A second PCR produced the PV57δ P1 with a 3′ nucleotide tail designed to be complementary to the 5′ sequence of TaV 2A. These two products then served to prime each other, in a third PCR, for 10 cycles prior to the addition of external primers. This PCR product then was inserted into pPink-HC using EcoRI and FseI (NEB). The RhPV IGR IRES (50) (GenBank accession no. AF022937.1, nucleotides 6576 to 7103) was synthesized (GeneART). The PV57δ-IRES-3CD also was introduced by overlapping PCR using the same steps as those described for PV57δ TaV 2A. All PCR steps were carried out with Phusion polymerase (NEB) using the manufacturer’s guidelines.

### Yeast transformation and induction.

Plasmids were linearized by AflII digestion and then transformed into PichiaPink strain one (Invitrogen) by electroporation. The transformed yeast cells were plated on *Pichia* adenine dropout (PAD) selection plates that lacked adenine and incubated at 28°C until sufficient numbers of white colonies appeared (3 to 5 days). To screen for high-expression clones, 8 colonies were randomly selected for small-scale expression experiments. Briefly, colonies were cultured in yeast extract-peptone-dextrose (YPD) for 48 h at 28°C with shaking at 250 rpm and then pelleted at 1,500 × *g*, resuspended in yeast extract-peptone-methanol (YPM; 0.5%, vol/vol, methanol) to induce protein expression, and cultured for a further 48 h. Cultures were fed methanol to 0.5% (vol/vol) 24 h postinduction. To determine the expression levels of each clone, the samples were analyzed by Western blotting as described below. For VLP production, a stab from a previously expressing glycerol stock was cultured for 48 h in YPD to high density. To increase biomass, 2 ml of the starter culture was added to 200 ml YPD in a 2-liter baffled flask and cultured at 28°C at 250 rpm for a further 24 h. Cells were pelleted at 1,500 × *g*, resuspended in 200 ml YPM (0.5%, vol/vol, methanol), and cultured for a further 48 h. Cultures were fed methanol to 0.5% (vol/vol) 24 h postinduction. After 48 h, cells were pelleted at 2,000 × *g* and resuspended in breaking buffer (50 mM sodium phosphate, 5% glycerol, 1 mM EDTA, pH 7.4) and frozen prior to processing.

### Sample preparation and Western blotting.

Cell-cultured virus supernatant samples were prepared through a 1:1 mixture with 2× Laemmli buffer and heated to 95°C for 3 min. For *Pichia*-derived protein samples, 100 μl culture was centrifuged at 2,000 × *g* for 5 min and resuspended in 100 μl 0.1 M NaOH for 5 min before being centrifuged at 2,000 × *g* for a further 5 min. Cell pellets then were resuspended in 2× Laemmli buffer and heated to 95°C for 3 min. Gradient fraction samples were prepared through a 1:5 mixture with 5× Laemmli buffer. Protein extracts were analyzed by 12% (wt/vol) SDS-PAGE using standard protocols. Western blot analyses were performed using a monoclonal primary antibody against VP1 protein (MAb 8560; Millipore), followed by detection with a goat anti-mouse secondary antibody conjugated to horseradish peroxidase, and developed using a chemiluminescent substrate (Promega). To identify viral protease 3CD, a rabbit polyclonal antibody was used, followed by detection with a goat anti-rabbit secondary antibody conjugated to horseradish peroxidase, and developed using a chemiluminescent substrate (Promega) (51).

### Purification of PV and PV VLPs.

Virus-infected HeLa cells were freeze-thawed and clarified by differential centrifugation. Supernatant was collected and virus pelleted through a 30% (wt/vol) sucrose cushion at 151,000 × *g* (using a Beckman SW 32 Ti rotor) for 3.5 h at 10°C. Virus pellet was resuspended in phosphate-buffered saline (PBS) and clarified by differential centrifugation. Supernatant was purified through 15 to 45% (wt/vol) sucrose density gradient by ultracentrifugation at 151,000 × *g* (using a Beckman SW 40 rotor) for 3 h at 10°C (14). *Pichia* cell suspensions were thawed and subjected to cell lysis using CF-1 cell disruptor at ∼275 MPa and chilled to 4°C following the addition of 0.1% Triton X-100. The resulting lysate then was centrifuged at 4,000 rpm to remove the larger cell debris, followed by a 10,000 × *g* spin to remove further insoluble material. The supernatant then was nuclease treated using 25 U/ml HS-nuclease (2BScientific) for 1.5 h at room temperature with gentle agitation. The supernatant then was mixed with saturated ammonium sulfate (40%, vol/vol) and incubated at 4°C overnight. The precipitated protein was pelleted at 4,000 rpm and resuspended in PBS plus 0.1% Triton X-100. The solution then was pelleted at 10,000 × *g* to remove any insoluble material. The supernatant was collected and pelleted through a 30% (wt/vol) sucrose cushion at 151,000 × *g* (using a Beckman SW 32 Ti rotor) for 3.5 h at 10°C. The resulting pellet was resuspended in PBS plus 0.1% Triton X-100 and clarified by centrifugation at 10,000 × *g*. Supernatant was purified through a 15 to 45% (wt/vol) sucrose density gradient by ultracentrifugation at 151,000 × *g* (using a Beckman SW 40 rotor) for 3 h at 10°C (14). Gradients were collected in 1-ml fractions from top to bottom and analyzed for the presence of VLPs through Western blotting and ELISA.

### ELISA.

A noncompetitive sandwich ELISA was used to measure PV-1 D and C antigen content (36). Briefly, 2-fold dilutions of antigen were captured using a PV-1-specific polyclonal antibody and then detected using PV-1-specific D antigen (MAb 234)- or C antigen (MAb 1588)-specific monoclonal antibody (kindly provided by NIBSC), followed by anti-mouse peroxidase conjugate (52, 53). BRP was used as the standard for D antigen content in each ELISA, which then was analyzed through a Biotek PowerWave XS2 plate reader.

### Electron microscopy.

To prepare samples for negative-stain transmission EM, carbon-coated 300-mesh copper grids (Agar Scientific, UK) were glow discharged in air at 10 mA for 30 s. Three-microliter aliquots of purified VLP/virus stocks were applied to the grids for 30 s, and then excess liquid was removed by blotting. To improve the concentration of particles on the grid surface, a second 3-μl aliquot was applied for a further 30 s, and then grids were washed twice with 10 μl distilled H_2_O. Grids were stained with 10 μl 1% uranyl acetate solution, which was promptly removed by blotting before another application of 10 μl 1% uranyl acetate solution for 30 s. Grids were subsequently blotted to leave a thin film of stain and then air dried. EM was performed using an FEI Tecnai F20 transmission electron microscope (operating at 200 kV with a field emission gun) with an FEI CETA CMOS charge-coupled device camera (Astbury Biostructure Laboratory, University of Leeds). Samples were imaged across a range of defocus values (−2.0 μm to −3.8 μm) at a nominal magnification of ×25,000, resulting in an object sampling of 0.418 nm/pixel.

### Image processing.

Raw micrographs were visualized with ImageJ, 1.51d (54, 55). The measuring tool of ImageJ was used to calculate VLP/virion diameter; three separate measurements were taken and averaged to give a diameter for each VLP/virion.

The RELION-3.0 pipeline (56–58) was used for further image processing of EM data. To generate templates for automated particle picking, a subset of particles was manually picked, extracted, and then classified by reference-free 2D classification. Automated particle picking resulted in large numbers of “junk” particles (corresponding to overlapping VLPs, disassembled VLPs, and other contaminants), which were removed by excluding poor-quality classes from a single round of reference-free 2D classification. Particles from the remaining classes were subjected to another round of reference-free 2D classification, and the resultant class averages were inspected. For each round of 2D classification, a regularization parameter of 2 was specified, and classification was allowed to proceed for 25 iterations. No symmetry was imposed during the 2D classification.
